# Unlocking the secrets of TGR5: a new dawn in treating diabetic cardiomyopathy

**DOI:** 10.52601/bpr.2024.240907

**Published:** 2024-08-31

**Authors:** Jin Li, He Huang

**Affiliations:** 1 Institute of Metabolism and Integrative Biology, State Key Laboratory of Genetic Engineering, School of Life Sciences and Zhongshan Hospital, Fudan University, Shanghai 200438, China

Diabetic cardiomyopathy, marked by dysregulation of lipid metabolism leading to cardiac remodeling and dysfunction, highlights the critical need for balancing fatty acid transport (Karwi *et al*. 2022; van de Weijer *et al*. 2011). CD36 stands as the predominant fatty acid transporter within cardiomyocytes, and its function is intricately tied to its expression and localization (Glatz and Luiken 2018; Shu *et al*. 2022). Despite recent progress in elucidating the mechanisms behind CD36's hyperactivity, strategies to manage its localization are still emerging.

Ming Xu from Peking University highlights the role of the bile acid receptor TGR5 as a regulator of fatty acid uptake. In groundbreaking research published in *Nature Metabolism*, Xu and his team reveal that activating TGR5 curtails cardiolipotoxicity and enhances cardiac function in diabetic mice, proposing it as a viable therapeutic target for diabetic cardiomyopathy (Wang *et al*. 2024). They discovered reduced bile acid levels in diabetic patients with myocardial injury and mouse models of diabetic cardiomyopathy, particularly those favoring TGR5 activation. The specific deletion of TGR5 in cardiomyocytes led to increased cardiolipotoxicity, aggravating cardiac remodeling and dysfunction in mice on a high-fat diet or with a diabetic *db*/*db* genotype (Fig. 1).

"The heart, a major fatty acid-utilizing organ, depends on the uptake and oxidation of these acids to regulate cardiac lipid metabolism", explains Ming Xu, co-corresponding author of the paper. "To unravel the mystery of TGR5 in cardiac lipid metabolism, it is critical to evaluate fatty acid uptake and oxidation". The team examined fatty acid uptake in TGR5-deficient hearts *in vivo* by intravenous administration of BODIPY fluorescent-conjugated fatty acids, and used the Seahorse Palmitate Oxidation Stress Test to assess exogenous palmitic acid oxidation in TGR5-deficient cardiomyocytes *in vitro*. They demonstrated that TGR5 deficiency increases long-chain fatty acid uptake and decreases oxidation, whereas TGR5 activation notably reduces heart fatty acid influx. “This is a promising target for regulating lipid metabolism in the heart”, said Hu Wang, the first author of the paper.

Further study into CD36, the chief mediator of long-chain fatty acid uptake, showed that TGR5 deletion doesn't affect CD36 protein levels but significantly increases its plasma membrane localization, enhancing fatty acid uptake. TGR5 activation, particularly through the INT-777 agonist, markedly reduces CD36's membrane presence. In addition, Src family kinases Fyn and Lyn assemble protein complexes on the plasma membrane to activate downstream signaling, while TGR5 inhibits the formation of the CD36/Fyn/Lyn complex. The team proposed that in TGR5-deficient cardiomyocytes, enhanced CD36 localization on the plasma membrane of cardiomyocytes could provide a plausible explanation for upregulated fatty acid uptake.

So how does TGR5 regulate CD36 localization on the plasma membrane? The researchers pointed out that post-translational modifications play important roles in CD36 localization, especially palmitoylation. Palmitoylation is a lipid modification that regulates the subcellular distribution and function of proteins by enhancing their lipophilicity (Linder and Deschenes 2007). Therefore, the level of CD36 palmitoylation determines its localization on the plasma membrane, which is consistent with previous studies. The team used an acyl-biotin exchange assay to detect the effect of TGR5 on CD36 palmitoylation. This study showed that TGR5 significantly inhibited the palmitoylation of CD36 in diabetic mice, thereby reducing its localization in the plasma membrane and cardiac fatty acid uptake. Protein palmitoylation is catalyzed by a family of palmitoyl acyltransferases. DHHC4, DHHC5 and DHHC6 participate in the palmitoylation of CD36 (Wang *et al*. 2019). DHHC4 and DHHC5 are mainly responsible for fatty acid uptake mediated by modified CD36, while DHHC6 is responsible for oxidized high-density lipoprotein. The authors demonstrate that TGR5 inhibits the interaction of CD36 with DHHC4 without affecting the binding of CD36 with DHHC5. Further, they knocked down DHHC4 in TGR5-deficient cardiomyocytes and found that DHHC4 knockdown inhibited CD36 palmitoylation mediated, suggesting that the effect of TGR5 on CD36 palmitoylation is DHHC4-dependent. The authors also confirmed that DHHC4 is a phosphorylated substrate for PKA downstream of G protein-coupled receptors, emphasizing that TGR5/DHHC4 signaling pathway mediates CD36 palmitoylation. This is the first study of palmitoylation in the field of cardiovascular disease and is eye-catching.

This paper presents a new mechanism for the regulation of cardiac fatty acid uptake. TGR5 deletion significantly enhanced cardiac fatty acid uptake, resulting in myocardial lipid aggregation, by promoting the localization of CD36 on the plasma membrane through the upregulation of CD36 palmitoylation mediated by the palmitoyl acyltransferase DHHC4. Researchers uncover a novel mechanism regulating cardiac fatty acid uptake via the TGR5/DHHC4 signaling pathway, emphasizing TGR5's therapeutic potential in diabetic cardiomyopathy management. "The collaboration was exceptionally fruitful, with each contributor playing a unique role", reflects Hu Wang. "Our systematic validation across various models not only confirms our findings but also bridges bile acid and cardiometabolic metabolism, opening avenues for future scientific exploration."

**Figure 1:**
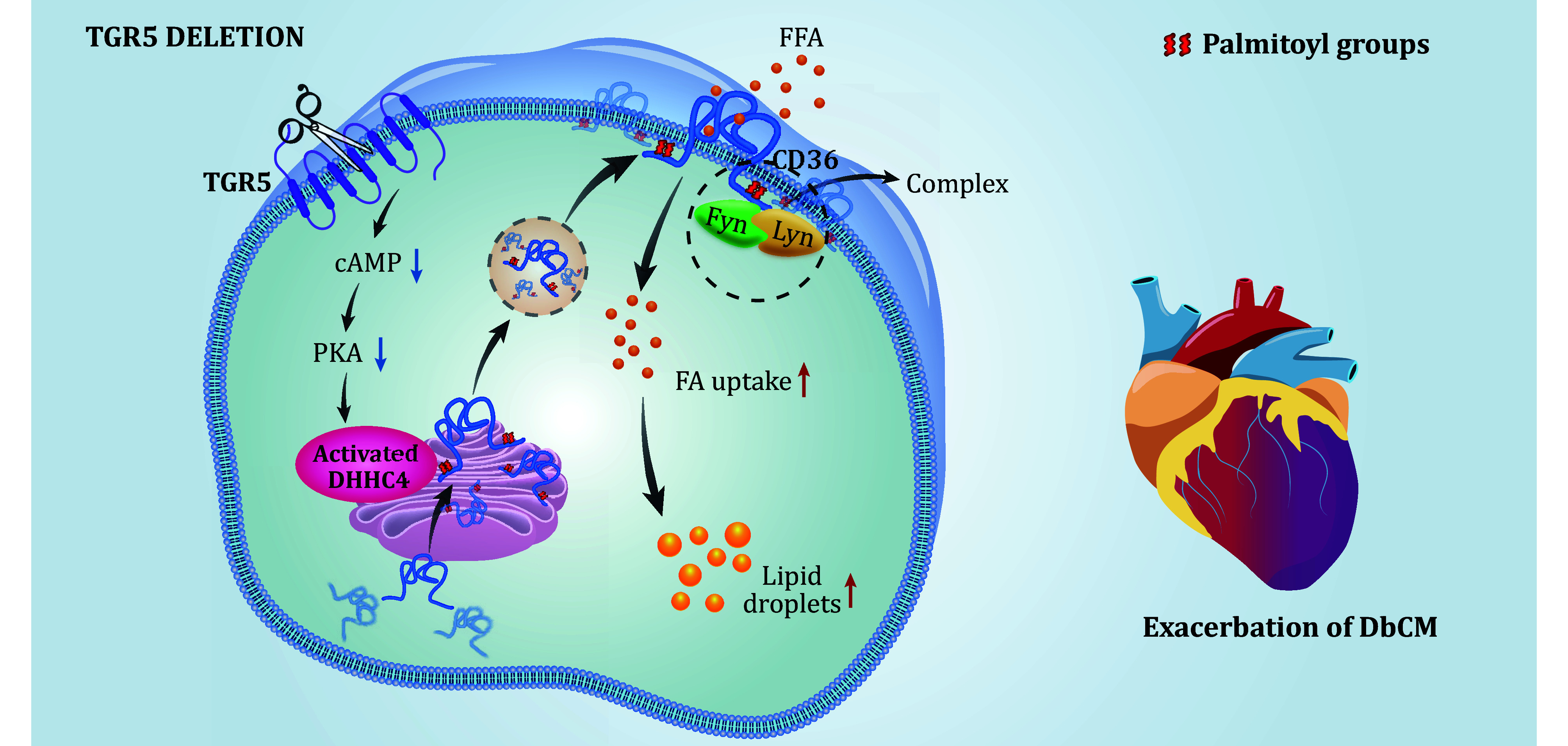
The graphical summary mediated by TGR5 in the regulation of DbCM (Wang *et al*. 2024)

## Conflict of interest

Jin Li and He Huang declare that they have no conflict of interest.
